# Public health measures during the COVID-19 pandemic through the lens of community organisations and networks in the Netherlands (2020–2021): five lessons for pandemic decision-making

**DOI:** 10.2807/1560-7917.ES.2022.27.42.2200242

**Published:** 2022-10-20

**Authors:** Carla Kolner, Wieke van der Borg, Jet Sanders, Jolanda Keijsers, Maysa Joosten, Marijn de Bruin

**Affiliations:** 1 Corona Behavioural Unit, National Institute for Public Health and the Environment (RIVM), Bilthoven, The Netherlands; 2 University of Humanistic Studies, Utrecht, the Netherlands; 3 Department of Psychological and Behavioural Science, London School of Economics and Political Science, London, United Kingdom; 4 Netherlands Organisation for Applied Scientific Research (TNO), Department of Healthy Living, Leiden, the Netherlands; 5 Radboud University Medical Center, Institute for Health Sciences, Nijmegen, the Netherlands

**Keywords:** COVID-19, adherence, community, organisations, key persons, pandemic preparedness, well-being, vulnerable people

## Abstract

**Background:**

During the coronavirus disease (COVID-19) pandemic, key persons who were formally or informally active in community organisations and networks, such as sports clubs or cultural, educational, day care and healthcare facilities, occupied a key position between governments and citizens. However, their experiences, the dilemmas they faced and the solutions they generated when implementing COVID-19 measures in their respective settings are understudied.

**Aim:**

We aimed to understand how key persons in different community organisations and networks experienced and responded to the COVID-19 measures in the Netherlands.

**Methods:**

Between October 2020 and December 2021, the Corona Behavioural Unit at the Dutch national public health institute, conducted qualitative research based on narratives derived from 65 in-depth interviews with 95 key persons from 32 organisations and networks in eight different sectors.

**Results:**

Firstly, key persons enhanced adherence and supported the resilience and well-being of people involved in their settings. Secondly, adherence was negatively affected where COVID-19 measures conflicted with important organisational goals and values. Thirdly, small changes and ambiguities in COVID-19 policy had substantial consequences, depending on the context. Fourthly, problem-solving was achieved through trial-and-error, peer support, co-creation and transparent communication. Lastly, the COVID-19 pandemic and measures highlighted inequalities in access to resources.

**Conclusion:**

Pandemic preparedness requires organisational and community preparedness and a multidisciplinary public health approach. Structural engagement of governments with key persons in community organisations and networks is key to enhance public trust and adherence to pandemic measures and contributes to health equity and the well-being of the people involved.

**Key public health message**

**What did you want to address in this study?**

In order to understand how key persons in different public and private service providers experienced and responded to the COVID-19 measures in the Netherlands, we performed interviews with 95 key persons from 32 different organisations in eight sectors.

**What have we learnt from this study?**

Key persons have an important role in enhancing adherence and supporting the well-being of people involved in their settings, but they also encounter conflicting goals and values. Trial-and-error, peer support, co-creation and transparent communication were employed to solve problems during the COVID-19 pandemic that obviously highlighted inequalities in access to resources in the Netherlands.

**What are the implications of your findings for public health?**

Pandemic preparedness requires organisational and community preparedness. Systematic engagement of governments with key persons in community organisations and networks, who often function as role models and have substantial impact on the behaviour of people within their context, is important to build public trust, to facilitate adherence to pandemic measures and enhance well-being.

## Introduction

Non-pharmacological public health interventions have been important in reducing the spread of severe acute respiratory syndrome coronavirus 2 (SARS-CoV-2) [1,2]. Behavioural research is often aimed at understanding individual citizen behaviours [3-5]. However, less attention has been paid to the behaviours and experiences of key persons (or ‘stakeholders’) in formal and informal community organisations and networks (public or private service providers), such as sports clubs, support groups, educational and cultural institutions or day care and healthcare facilities. These key persons occupy a key position between the national government, public health authorities and citizens and faced the challenge of putting national coronavirus disease (COVID-19) measures into practice while safeguarding the health and well-being of the people involved in their settings. Hence, their role seems potentially critical to finding an effective response to the pandemic, supporting the well-being of the population and ensuring long-term support for pandemic measures.

Early in the pandemic, the World Health Organization (WHO) published a guideline [6] to promote community engagement to prevent and contain COVID-19. The WHO describes the community as an active rather than a passive actor in the containment of a pandemic and trust building as the central element to encourage people to comply with public health regulations. Targets of community engagement include strengthening existing and new partnerships, strengthening community governance structures to build capacity among stakeholders, and empowering and supporting organisations and communities with regard to COVID-19 response efforts. Another important aspect is the systematic engagement with vulnerable groups, such as people with disabilities and migrants, to ensure measures and communication are tailored to their needs and challenges. In this way, community engagement serves social justice and enhances health equity as well.

**Figure 1:**
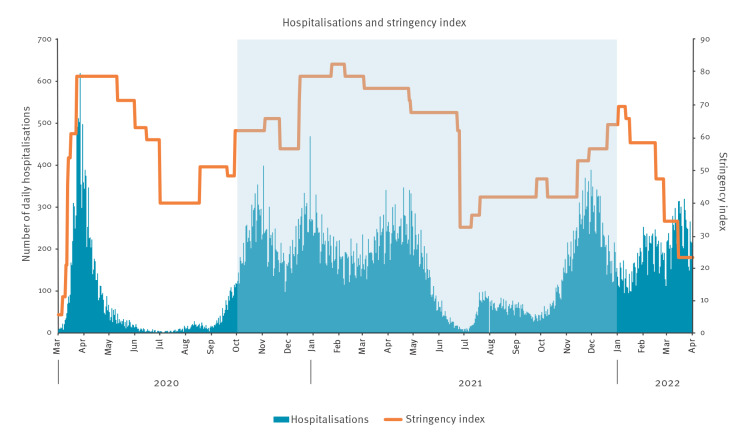
Hospitalisations with COVID-19 and stringency index, the Netherlands, March 2020–April 2022

Source: National Institute of Public Health and the Environment of the Netherlands [19] (hospitalisations) and Oxford COVID-19 Government Response Tracker [20] (stringency index). The stringency index is a mean score of the stringency level of a government’s response to control coronavirus infections, ranging from 0 (no measures) to 100 (strictest measures).

In line with the WHO guidelines, Michener et al. give many examples of community engagement during the COVID-19 pandemic [7]. Among others, they mention the value of peer support, the help of involved residents who build a bridge between healthcare agencies and their respective communities, and the role of community and partnerships in helping and guiding people to comply with COVID-19 measures [7]. However, we did not find any study that focuses specifically on the experiences and values of key persons during the COVID-19 pandemic or on their needs in order to fulfil and optimise their role. In order to better understand and meet the needs of these key persons during future outbreaks, we conducted a qualitative study to examine how a broad range of key persons in community organisations and networks experienced and responded to the COVID-19 measures over time in the Netherlands. For an overview of COVID-19 restrictions in the Netherlands (comparable to those in most European countries), see Figure 1 for hospitalisations, the stringency index and basic measures.

This study was conducted in the period from October 2020 until December 2021, highlighted in light blue in the figure.

Basic COVID-19 measures in the Netherlands: in case of symptoms, stay at home and get yourself tested; maintain a distance of at least 1.5 m from others; regularly wash your hands and cough or sneeze into the crook of your elbow; work from home as much as possible; avoid busy places and congestion; leave when it's too busy; try to travel during off-peak hours; wear a non-medical face mask in all public indoor spaces (see time line) [21].

## Methods

### Research team and reflexivity

The research team consisted of six researchers/interviewers (hosts) with a health or behavioural sciences background and four research assistants (observers). All six interviewers followed formal university training in qualitative research and were experienced in conducting qualitative interviews. Three researchers involved in the coding and thematic analysis of qualitative data and the use of relevant software (MAXQDA version 2022, Verbi, Germany) were trained for this purpose and had applied these skills in previous qualitative studies. For further quality assurance, we invited four members from our institute’s independent scientific advisory board and four independent field experts to form an expert panel (see acknowledgements). This panel gave input on the study design [8-10], gave feedback during all steps in the process and reviewed the analysis and conclusions.

### Study design and procedure

**Figure 2:**
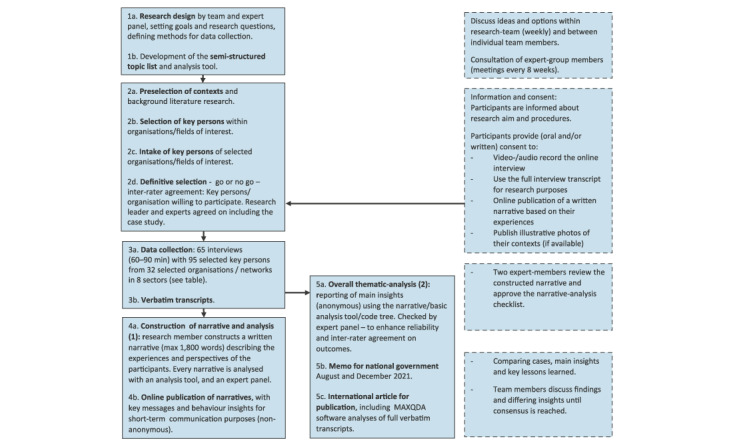
Schematic overview of study design and procedures

We opted for a narrative approach with video-recorded, in-depth interviews with representatives and key persons of a broad selection of sectors, organisations and networks in different communities in the Netherlands (Figure 2, steps 1–5 for the study procedure). With regard to the narrative approach, we refer to the definition of Czarniawska [11]: ”*a spoken or written text giving account of an event/actions or series of events or actions, chronologically connected and were people informed us about their intentions, purposes, interactions with others and reflect about the meaning of their actions or behaviour in a certain period of time”.* For this study, we focused on COVID-19-related experiences. We used the Dutch COVID-19 Prevention Behaviour Model [12,13] as the theoretical framework to help identify the key factors and behavioural determinants, such as risk perceptions, social and motivational processes, barriers, and problem-solving activities to enhance adherence to COVID-19 measures and maintain well-being.

MAXDQA: MAXQDA software analysis version 2022, Verbi, Germany.

### Materials

We developed a semi-structured topic list (step 1b, Supplement S1) and pretested it in three pilot interviews. The topic list helped us to encourage respondents to reconstruct – retrospectively and chronologically – their experiences with and the impact of COVID-19 measures in their community or organisation, both for themselves and what they observed for others. Examples of questions include: ”*What happened when COVID-19 measures were implemented in your context?”*, ”*What facilitated or hindered adherence?”*, ”*Which dilemmas were faced?”*, “*How did people cope with these dilemmas?”*, “*How were work processes or partnerships affected or adapted?”*, “*What were the main themes in discussions?”* and “*What worked in your context with your employees, pupils or clients?”* For analysis purposes, we used the topic list to create an analysis tool (i.e. a code tree, see Supplement S2) to capture the crucial information for each case such as key issues, dilemmas, solutions and workable ingredients.

### Participant recruitment

As a form of purposeful sampling [14] we focused explicitly on a variety of key persons in eight sectors in 32 different contexts (Table). We aimed to capture multiple perspectives and varied experiences and views on the implementation of COVID-19 measures. We recruited organisations and networks that were affected by the pandemic policies (e.g. through closures or other measures) and are important for social life, with specific attention to organisations and networks for people in a physically, socially or mentally more vulnerable position (e.g. day care for homeless people, youth work, healthcare facilities for the elderly or people with disabilities, support networks for migrants or the lesbian, gay, bisexual, transgender, queer, intersexual (LGBTQI) population). These groups are often underrepresented in research.

**Table:** Organisations/networks, sectors, and respondent characteristics per narrative, date of interviews, the Netherlands, September 2020–November 2021

Context/organisation – network	Sector	Respondents (n = 95)	Interview date
1. Primary school	Education	Teachers and head of school (n = 3)	Apr 2021
2. Vocational school	Education	Teachers and team leader (n = 3)	Jan 2021
3. Student accommodation	Education	Students (aged 20–25 years) living together (n = 3)	Dec 2020
4. University	Education	Communication science students and professionals (n = 3)	Oct–Dec 2020
5. Funeral home/ undertaker	Entrepreneurs	Attendants, entrepreneurs (n = 2)	Dec 2020
6. Events sector	Entrepreneurs	Manager of climate event and experiential expert/entrepreneur (n = 1)	Jan–Feb 2021
7. Young entrepreneurs	Entrepreneurs	Young entrepreneurs in the fashion industry (n = 2)	Mar 2021
8. Hotel/restaurant/catering	Entrepreneurs	Hotel chain owner/manager and solo caterer (n = 2)	Jul 2021
9. Municipality – shop owners	Public space	Policymaker, owners of shops and restaurants in the city (n = 3)	Sep 2020
10. Municipality – enforcement service	Public space	Enforcement officers and community- service/policy control/inspection employee (n = 5)	Jul–Aug 2021
11. Free food market	Neighbourhood	Civic initiator, social workers in deprived area, lecturer at a university of applied sciences (n = 5)	Feb 2021
12. Day- and night care for homeless people	Neighbourhood	Crisis manager, caregiver, lecturer at a university of applied sciences, sector representative (n = 4)	May 2021
13. Mediation and cohesion	Neighbourhood	Mediators/volunteers, lecturer at a university of applied sciences (n = 5)	May–Jun 2021
14. Police and ambulance service	Formal care and safety	Police officer and ambulance personnel (n = 3)	May 2021
15. Home for the elderly	Formal care (elderly people)	Family caregiver, day care assistant, lecturer at a university of applied sciences (n = 3)	Dec 2020
16. Home care professionals	Formal care (elderly people)	Home caretakers and family of a severe ill person (n = 2)	Aug 2021
17. Day care for developmentally disabled people	Formal care (mental health)	Day care professionals and policy and quality officer (n = 2)	Dec 2020
18. Mental health sheltered living organisation	Formal care (mental health)	Board member, social worker, quality and communication employee (n = 3)	Sep 2020 Dec 2020 Jan 2021
19. Hospital/COVID-19 unit	Formal care (COVID-19-related)	Nurses working in COVID-19 intensive care and cohort units (n = 3)	Jul 2021 Oct 2021
20. Informal family care	Informal care (elderly people)	Family caregivers of a 99-year-old vulnerable person (n = 2)	Dec 2020
21. Home care for people with chronic disease	Informal care (adolescents))	Caregivers – patients with chronic disease (n = 2)	Nov 2020
22. Youth care/home safety	Work/formal youth care and safety	Professional /caregiver (n = 2)	Jul 2021
23. Youth community worker	Work/social youth work	Social workers, alderman (n = 4)	Feb 2021
24. Child day care (ages 0–4 years)	Work/child day care ages 0–4 years	Childcare employees, managers, parent (n = 3)	Aug 2021 Nov 2021
25. Women – ethnic groups	Sport	Initiators of civil sport initiative, attendants (n = 8)	Nov 2020
26. Sports clubs	Sport	Football trainers and swimming clubs/sector representatives (n = 4)	Apr­–May 2021
27. Movement stimulation	Sport	Sports coach and walking challenge initiator (n = 2)	Jan–Feb 2021
28. Amateur music company	Culture (music)	Board member/musician, sector representatives (n = 3)	Nov 2020–Jan 2021
29. Museum	Culture (arts)	Director, education and communication professional (n = 2)	May 2021
30. Performance business	Culture (performers)	Performer/artist and visitor (n = 2)	May 2021
31. Film industry	Culture (film)	Film producer, COVID-19 set manager (n = 2)	Jul 2021
32. LGBTQI communities and refugee arts groups	Culture (vulnerable groups)	Initiators of self-care organisations and LGBTQI and refugee communities experiential experts (n = 2)	Jun 2021

COVID-19: coronavirus disease; f: female; m: male. LGBTQI: lesbian, gay, bisexual, transgender, queer, intersex.

For selection and preparation purposes (steps 2a–d), we conducted background literature searches on the impact of COVID-19 in that particular context. We contacted representatives of community networks and various organisations within each sector using a snowball sampling strategy. We identified possible respondents by contacting sector-relevant organisations, field experts and knowledge institutions. We conducted intake interviews with all potential respondents to make sure they met our main criteria (experience – priority was given to persons with experience in their sector, but if the respondent did not have much experience a manager was also interviewed, representation of an affected sector/target group, involved in or affected by COVID-19 measures, consenting to participate).

We informed each participant and organisation about the aims and methodology of the study, both orally and in writing. We obtained both written and audio-/video-recorded consent for participation and publication. Once all sides (research group, expert panel, organisations and respondents) had given their agreement, we made a decision whether to include a participant or organisation (go or no go). In total, we conducted 65 interviews with 95 stakeholders from the 32 selected contexts.

### Data collection

To enhance the validity, reliability and credibility of the study [8-11] we aimed to study multiple perspectives in each case. Depending on the context, we interviewed between two and five key persons per case for 60–90 min each (step 3a). To understand the impact of policy changes over time, we spread out the interviews with the different key persons over a period of 15 months (October 2020–December 2021). Due to COVID-19 restrictions, we only collected data online and asked respondents to send illustrative images of their environment or work setting. All interviews were conducted by one interviewer and one observer, audio-/video-recorded and transcribed verbatim (step 3b). We stopped interviewing when no new themes or insights emerged and data saturation was reached [10].

#### Analysis 1: development and analysis of the narrative

For the purpose of communicating important insights to the general public, sectors and professionals, we constructed narratives that covered key issues and lessons learned per case and published them ([15], Figure 2 and steps 4a and 4b, Supplement S3 and S4). The interviewers and observers developed the narrative iteratively and in co-creation with the respondents. Involvement of the respondents prevented misinterpretation and clarified the relationship between key determinants and stakeholders’ behaviour in that context. After a member/error check by all respondents [9] the narratives were reviewed by at least two members of the expert panel. Due to sudden developments in the pandemic and the implementation of new COVID-19 measures, this sometimes led to additional interviews with the same respondents. Finally, we analysed every narrative, which had a high information density, with the analysis tool and listed the key themes and findings. To increase inter-rater reliability, we discussed discrepancies and different interpretations (e.g. about workable ingredients) with two members of the expert panel and (if necessary) with the respondents until a consensus was reached.

#### Analysis 2: overall thematic analysis

For the purpose of rapid feedback for policymakers, the research team conducted an inductive thematic analysis of all narratives [14,16]. We used the analysis tool to look for patterns (Figure 2, step 5a). Using MAXQDA software, we searched for deeper insights, illustrative practices and quotations. After review by the research team and the expert panel, we reported the overall analysis and findings to the Ministry of Health, Welfare and Sport [17] (Step 5b) and incorporated them into the current article. The interviews and analysis were performed in Dutch. Quotes obtained were then translated to English for the purpose of displaying these results.

We followed the Consolidated criteria for reporting qualitative research (COREQ) guidelines for the reporting of this study [8].

## Results

Five main insights emerged in the course of our research (Box 1) as well as four categories with several themes (Box 2).

Box 1 **Five main insights**

- Formal and informal key persons played a crucial role in promoting adherence and strengthening the organisational resilience and well-being of people involved in their settings
- Adherence was affected where COVID-19 measures conflicted with organisational goals and values
- Small changes, ambiguities and uncertainty in COVID-19 policy had substantial consequences, depending on the context
- Problem-solving was achieved through trial-and-error, peer support, cocreation and transparent communication
- The COVID-19 pandemic and measures highlighted inequalities in access to resources

Box 2 **Categories and themes**

**Dilemmas**

Health of self vs health of others;
Protection from COVID-19 vs social and mental health or safety;
Adherence to measures vs quality of work and care (conflicting goals, values, identities).

**Tensions**

Ambiguity of policies/communication (uncertainty, continuous changes);
Unfeasibility of measures (not being able to comply to measures because they are perceived as unjust, incomprehensible, unpractical because of a lack of time or space for implementation);
Physical and social distance;
Confusion of role and responsibilities (autonomy and identity).

**Solutions**

Enhancing social cohesion/connection (by giving voice, keeping people connected one way or the other, enhancing collaborative reflection, creating mutual understanding, enhancing involvement - organising outdoor activities, using social media and technical/online solutions);
Transparent communication (by providing context specific, tailor made information using trustworthy channels, clarification or translation of measures, making measures understandable and logic, refuting misinformation);
Tailor made adjustments/context-specific protocols, setting or reaffirming social norms in context;
Adapting or revising work processes, reprogramming (finding a new equilibrium or balance together, rethinking meaning, innovation);
Providing example behaviour/role modelling, guiding, explaining;
Stimulation of cooperation (horizontal, vertical - inside organisation);
Starting new organisational partnerships, developing stronger networks (outside organisation);
Facilitating dialogue and co-creation, listening to the needs of people (showing and stimulating empathy and compassion);
Providing (peer) support (moral, social, mental);
Providing practical/financial support, access to services;
Deliberately taking calculated risks to promote or preserve well-being.

**Workable ingredients**

Connection and belonging, social cohesion, engagement;
Empowerment (e.g. being better informed, enhanced capacity or skills), social or financial security and inclusion;
Improved coping mechanism, such as accepting dynamics by default and uncertainty, re-finding meaning, resilience;
Being able to talk together – sharing feelings (preventing fear or panic);
Acknowledgement of emotions, attitudes and beliefs, feeling part of a group or community;
Collaboration, knowledge exchange, enhance mutual trust, reinforced solidarity, combining powers of formal and informal key persons;
Autonomy to make decisions and taking responsibility;
Trial and error, learning by doing creativity, flexibility.

### Formal and informal key persons played a crucial role in promoting adherence and strengthening the organisational resilience and well-being of people involved in their settings

We noted many tailor made adjustments in and outside organisations. Both professionals and laypersons supported and empowered others to cope with the pandemic policies in many ways. For example, they constantly adapted COVID-19 measures to fit their context and the environment to facilitate their implementation. Organisations customised work processes, mitigated negative side effects (like fear or panic), translated information and refuted misinformation. Formal as well as informal stakeholders functioned as role models. Their support served different purposes (morally, socially, mentally and financially) and had a positive impact on adherence as well as on social cohesion and well-being. This was often due to the acknowledgement of problems or tensions people experienced, activities aimed at keeping people connected one way or the other and dialogue that encompassed knowledge, emotions, beliefs and attitudes.

”*A weekly digital gathering was organised, like a minor press conference… Our director, but also people from elsewhere within the organisation could contribute. Recent COVID-19 measures were generally clarified and translated to the more specific context of our museum… Furthermore, courses to ensure employees’ mental resilience were offered… By those means we strove to remain cool, calm and collected”* (museum employee).

Organisations also started new partnerships with other organisations to develop stronger networks for cooperation and knowledge exchange. For example, shop and restaurant owners and a municipality formed a new communication platform to be better able to cope with the unpredictable impacts of new policies on life and business in the city centre. Similarly, reinforcement officers in public spaces intensified their cooperation with police and youth work professionals in the community to prevent overcrowding and youth-related problems (e.g. adolescents violating curfew). And in a poor neighbourhood, active citizens and volunteers worked together with social workers, religious leaders and the municipality to restart a food bank that had been closed at the beginning of the COVID-19 pandemic. These forms of cooperation improved the exchange of knowledge and reinforced solidarity and mutual trust between partners.

### Adherence was affected where COVID-19 measures conflicted with organisational goals and values

We observed that risk perception extended beyond being aware of the risk of COVID-19, it also included social and mental vulnerability.

*”On the one hand, you have vulnerable groups from a medical point of view… On the other hand, there are socially vulnerable groups who don’t have mental resilience and for whom contact is extremely important. For one person, having no contact is safer, but for the other… not being in contact can be deadly”* (community leader).

Measures with a high social impact, such as physical distancing, visitor restrictions and face mask policies, were especially felt as a hinder to communication and connection between staff and with clients, children/youngsters, parents etc. This affected the quality of life, work and care. Respondents reported many dilemmas and problems to be solved in order to adhere to COVID-19 measures, especially when these measures conflicted with work-related goals, values and identity. For example, nursing home staff reported difficulties in taking care of elderly people with dementia. They reported increased loneliness as well as confusion and anxiety in patients due to missed family care and caregivers wearing face masks. Similarly, youth care professionals reported deliberately entering homes or violating other COVID-19 measures to make sure that children were safe and to protect them from abuse or violence in the home. Funeral attendants occasionally neglected measures when comforting grieving relatives. These conflicting values and goals were mentioned as reasons for deviating from the rules deliberately. They were seen as a trade-off between the conflicting goals of safeguarding the well-being of the organisation/person and managing COVID-19 risks.

### Small changes, ambiguities and uncertainty in COVID-19 policy had substantial consequences, depending on the context

Each policy adjustment led to a cascade of activities for organisations trying to comply financially, practically and emotionally. Even small changes had substantial consequences, especially in complex contexts. In one example, an unanticipated extension of a lockdown combined with a curfew required a round-the-clock care service for homeless people to be able to suddenly arrange furnished emergency housing (e.g. empty offices and hotels) for every homeless person in the country within 3 weeks. In another example, small changes in permitted group size for social events, sports, classrooms etc. and a lack of clarity about the duration of this policy caused substantial stress for event organisers, sports trainers, primary school staff, volunteers, pupils and parents. This was because each small change necessitated the adjustment of all schedules, work and communication processes. Ambiguities and uncertainty with regard to restrictions were especially difficult to cope with, not only for key persons and employees in organisations but also for entrepreneurs.

*”I had a conversation with a client who is an entrepreneur in the events sector. He supplies tables, chairs etc. for events. That sort of stuff. … He merely sits at home now and he is mentally a wreck. He tried everything and attempted to think out of the box, but because of constantly changing policies he cannot find a proper plan for his business”* (community leader in a poor neighbourhood).

Adherence to measures required an ongoing search for tailor made solutions. This had a serious impact on key person, employee and client well-being, especially as measures kept changing over time.

### Problem-solving was achieved through trial-and-error, peer support, co-creation and transparent communication

In times of emergencies, daily routines are often disturbed. The key in such cases is to restore balance by finding new routines. Because of the unpredictability of constantly changing measures during the COVID-19 pandemic, organisations often relied on ‘trial-and-error’ and ‘learning by doing’ to develop practical solutions for implementing measures in their settings. These solutions evolved over time until a workable equilibrium was achieved. Stimulating continuous, transparent communication and dialogue (i.e. explaining the reasons for measures, showing empathy and compassion for difficulties people experienced and stressing positive messages as well) and involving all members of the organisation in addressing challenges strengthened organisational creativity and innovation. Promoting co-creation and cooperation between colleagues, as a form of informal peer support and involvement and co-creation between key persons on different organisational levels, such as policymakers, staff and co-workers, facilitated workable solutions. Collaborative reflection on the reason for and impact of the COVID-19 pandemic and measures was especially helpful to reaffirm social norms and shared values in their settings, generate energy, reinforce creative problem-solving and enhance adherence. For example, nurses in COVID-19 units in hospitals supported each other every day during or after work in through digital group communication (using apps) and wanted to be involved in the decision making process. In their opinion, this was crucial to cope with all the tensions and stress and provide effective solutions for problems they faced.

“*I think that it is most important that our superiors always keep communicating with us… because we take care of the COVID-19 patients. It’s so important that we have a say and influence and are involved in the decision making process. I think this is one of the most important things… to be involved in everywhere”* (COVID-19 unit nurse).

In the sports and cultural sectors, multiple months of lockdown resulted in innovative ideas for reorganisation, renovation and adjustments to promote employee well-being. A lack of customer influx stimulated a rethink of the meaning or organisations in society and a revision of work processes, such as providing sports training outside (balcony gym for elderly people), opening up empty hotel rooms for flexible working arrangements and for homeless people, moving museum exhibitions outside, finding new target audiences online and adopting a ‘dynamic by default’ mindset for the future.

### The COVID-19 pandemic and measures highlighted inequalities in access to resources

One effect of the pandemic was that inequalities between people in terms of access to resources, and shortcomings in public services and organisations designed to reach or support vulnerable people became more obvious and compounded other issues. For example, information about new COVID-19 measures communicated through press conferences on national television and in accompanying written and online information often did not reach people who had problems with understanding Dutch or interpreting the measures.

”*One important factor among this group that does not take the measures seriously is that these people are used to a complex existence in poverty that is full of problems, their heads are full… At that point, a virus doesn’t make much difference and they won’t let it scare them off”* (social worker in a poor neighbourhood).

People who already suffered from loneliness (e.g. elderly people, people with disabilities or chronic disease, asylum seekers) became much more vulnerable because of the physical distancing requirements and visitor limitations and often felt ashamed to talk about it.

Key persons in networks of minority groups, such as migrants or members of the LGBTQI art community, are close to their members and able to signal problems at an early stage. They reported several shortcomings in the financial, emotional and social support for these groups and observed members withdrawing from services and society. Cooperation between formal (i.e. social/youth workers) and informal key persons (i.e. volunteers, community leaders or influencers) in local community organisations and networks seemed crucial to combine powers and to activate community infrastructures and trustworthy channels to reach marginalised groups, build trust and support adherence to COVID-19 measures.

## Discussion

In line with the WHO declaration on community engagement [6], the results of this study demonstrate the important and active role of organisations and communities during the COVID-19 pandemic. They shed light on the responsibilities that key persons in organisations and communities assumed almost automatically to protect the health and well-being of employees and fellow citizens, and promote the implementation of COVID-19 measures in their settings. Our results also accord with other studies insofar as they highlight the relevance of context, community resilience and social cohesion [7,18]. They also emphasise the importance of building on community strength and priorities and mention multiple reinforcing factors (e.g. engagement, culturally and linguistically appropriate health risk messaging, co-creation, partnerships and trust) in shaping, communicating, implementing and disseminating public health strategies [7]. As in many other countries, the focus of pandemic policymaking in the Netherlands was medical, and included protecting vulnerable people and avoiding overloading the healthcare system. Many of our respondents critically pleaded for greater attention and consideration for the quality of social relationships, which are strongly related to well-being, quality of life, work and care. With this in mind, what can policymakers, communication departments and organisations in the public health and social domains – at all levels – learn from this study when it comes to planning for future outbreaks or pandemics? 

These results show the importance of involving and supporting key persons, focusing on transparency and building trust, sharing lessons learned between the various organisations and key persons in these organisations and networks, supporting cross-discipline and intersectoral collaboration as well as to promoting community and organisation preparedness to enhance equity.

Key persons in organisations and communities developed a multitude of coping strategies to support people in complying with COVID-19 policy, while safeguarding well-being by promoting and restoring social cohesion and inclusion. Structural and active involvement of them as key partners in an early stage of the planning of COVID-19 prevention policies, (e.g. by encouraging them to make sector or community plans, or by forming an advisory group that represents various organisations and stakeholders) is important to identify their needs in time and enhance trust and adherence to COVID policy.

It is furthermore, important to support key persons morally, practically or financially in the multiple challenges they face to maintain a good balance between infection prevention and well-being, quality of life, work and care. There are many lessons to be learned from this group if contact is maintained during smaller scale outbreaks (e.g. when the well-being of vulnerable groups is at stake). Key persons will also profit from more time and guidance in the implementation process.

Using transparent, clear and pretested communication and implementation strategies, with respect to diversity in communities, explaining the reasons and necessity of restrictions and focussing not only on restrictions but also on opportunities and desired courses of action is likely to enhance acceptance and to promote trust and adherence [7]. Combining the powers of formal and informal stakeholders in community networks can activate existing communication methods using trustworthy channels that are better attuned to the needs of vulnerable groups.

Key persons learned through trial-and-error how to deal with restrictions and dilemmas in a range of settings, resulting in increased resilience. Proactively encouraging and facilitating them to share lessons learned within and between organisations, at all levels, can benefit organisations during a pandemic. It may also benefit the viability of organisations after the pandemic, expedite pandemic recovery and enhance preparedness.

In a pandemic, people can easily become more vulnerable, and new vulnerable groups emerge. Meanwhile, relevant information or social or financial support may not reach the groups that need it the most. As a consequence, communities and organisations need to be prepared at an early stage to track and monitor vulnerable people and support them according to their needs with the help of social and community experts.

A limitation of this study concerns possible limited credibility. Because of the social restrictions, we were not able to make observations on the spot and we could not check the credibility of the narrative. The knowledge that the narrative would be published online may also have led to more socially desirable answers. We tried to prevent this by emphasising our neutrality, encouraging respondents to talk about positive as well as negative experiences and issues they faced, collecting multiple perspectives, asking for pictures and other relevant documentation, and making explicit to our respondents that sensitive information would be anonymised in reports or articles. Another limitation concerns the possible selection bias, since not all key persons, representatives, contexts or sectors could be included. For example, organisations with a strongly negative attitude towards measures were not explicitly recruited for this study. Nevertheless, the main lessons learned here were evident across the wide range of cases and interviews we conducted.

### Conclusion

In this study, we gave voice to multiple formal and informal key persons from community organisations and networks about their contribution to containing the COVID-19 pandemic. We conclude that these key persons played a crucial role in implementing pandemic policies in society and enhancing health equity and well-being. It thus appears key that these organisations are actively supported by the local and national government and fully acknowledged as key partners in a multidisciplinary pandemic control approach.

## Supplementary Data

348000 Supplementary Material
