# Supplementary material for: Public health measures during the COVID-19 pandemic through the lens of community organisations and networks in the Netherlands (2020–2021): five lessons for pandemic decision-making
Source: Euro Surveill. 2022 Oct 20;27(42):2200242. doi: 10.2807/1560-7917.ES.2022.27.42.2200242 (PMC9585880; doi:10.2807/1560-7917.ES.2022.27.42.2200242)
Supplement: Supplementary Material [file 22-00242_KOLNER_Supplementary_material.pdf]

This supplementary material is hosted by *Eurosurveillance* as supporting information alongside the article 'Public health measures during the COVID-19 pandemic through the lens of community organisations and networks in the Netherlands (2020–2021): five lessons for pandemic decision-making', on behalf of the authors, who remain responsible for the accuracy and appropriateness of the content. The same standards for ethics, copyright, attributions and permissions as for the article apply. Supplements are not edited by *Eurosurveillance* and the journal is not responsible for the maintenance of any links or email addresses provided therein.

Content:

1. **Semi structured questionnaire**
2. **Analysis tool/checklist or code tree**
3. **Definition of a narrative**
4. **Translated narrative and background info: Free Food Market in or out of the shadows**

Supplement S1. Semi structured questionnaire

**Content format/interview form**<br>**Name of example:**<br>Short explanation (max 30 words)<br>**Organisation/ place (municipality):**<br>Which organisation has put this example into practice? Which area?<br>**Sector:**<br>Which sector/theme does the example belong to?	
1. **Name and job title of contact person**	Who is the contact person? What is the person's job title or position in the organisation?<br><br>Contact details for other people... (e.g. communications consultant)
2. **Target group**	What is the target group for the interventions? Why is this target group interesting from the perspective of infectious disease control? Does it involve a vulnerable target group with additional risks?
3. **Which basic measures apply?**	What COVID-19 behavioural rules (distancing, avoiding crowds, quarantine, testing, rapid testing, etc.) are (in principle) required in this setting? (e.g. face masks, closing at 22:00 hrs, or maximum group size)

<table>
<tr>
<td>4. Description of the setting and context, focus on risk-perception and contextual factors, protocols, specify targetgroup etc.

Invite people to tell their story chronologically and retrospectively</td>
<td>General description of setting and context:

- Who is involved here?
- What risks are involved here and how have they been assessed? (Was a risk analysis performed?)
- What factors are involved other than COVID-19? (Consider aspects that affect quality of life.)
- What makes this environment or setting interesting/exceptional?
- What protocols apply here?
- Who are the individuals who are most directly involved? (Who would be interesting to interview? Check!)

Frame the narrative from the point in time that COVID-19 arrived. What happened? Who took action?

Find out what story absolutely has to be told by the person submitting the example. What is on the tip of the tongue? What feels most urgent? In the sense of: what have people in that setting already been through in terms of COVID-19 and the coronavirus measures, what is behind them, and what is relevant right now in the current situation? Don’t just focus only on difficulties, but ask more in-depth questions about the beautiful experiences.</td>
</tr>
<tr>
<td>5. A closer look at the target group, focusing on the behaviour and vision behind this example, the dilemma’s, concerns for wellbeing, coping mechanisms, obstacles etc</td>
<td>Which target behaviours are the focus of this example and which particularly relevant factors influence the behaviour of the target group?
- What are the particular characteristics of the target group that need to be taken into account?

- How was C-policy (i.e. the interventions) established and rolled out?

- What approach was used in this example?

- What factors, struggles or dilemmas present in the context made it particularly difficult to follow the measures in this context, and maintain compliance in the longer term? (And which measures, in principle?) And what larger considerations were involved here?

- What were main themes in discussions?

- Is there an explicit focus on employee well-being and quality of life? What aspects were specifically addressed?

- Is the target group involved in coming up with solutions to find an (new) equilibrium? If so, how?

- How have people handled opportunities to support the measures and/or address the obstacles and dilemmas?</td>
</tr>
</table>

**6. Which interventions or solutions/ behavioural alternatives apply?**	Description of the targeted or non-targeted interventions or courses of action and activities (or some combination thereof) to promote compliance and to restore the balance in the context:<br>- How do people try to increase compliance with the coronavirus behavioural rules?<br>- Similarly, how do people try to support maintaining the behaviour in the longer term?<br>- Was there a special reason to do so? (besides COVID-19 on its own)<br>- How have people tried to minimise loss of well-being or quality of life, to compensate for loss or to overcome resistance?<br>- Has the organisation developed special activities that may be interesting to others?
7. **Methodology/ determinants**	Which mechanisms and techniques are in principle used or become apparent in order to influence the behaviour intended under point 5, and via which determinants? Cf. Skinner's behavioural model.<br>- Examples: nudging, empathy, social norm, modelling etc. but also various coping styles or strategies<br>- Cues to action<br>- Self-regulation<br>- Changes in habitual behaviour/rituals - Changes in the environment - Cooperation……..<br>...to facilitate desired behaviour?
8. **Assessment of effectiveness as presented by the organisation, workable solutions and ingredients, partnerships**	What experiences has the organisation had with the solutions/interventions?<br>What worked well in their situation and target group to cope with the measures? What didn't?<br>Why does the organisation believe that chosen solutions were/are effective?<br>Is the approach based on a substantiated method, or is it more about learning by doing, or trial and error...<br>What active workable ingredients are involved, according to the person submitting the example? Do not just look at separate interventions, but also consider the integrated approach.<br>Did stakeholders formed (new) partnerships to be better able to cope….?
9. **Specific considerations about implementation of the programme**	What is important for proper implementation of the interventions?<br>Does/did the organisation encounter difficulties?<br>What would they do differently next time?<br>Are there specific tips and tricks that would be characteristic of this situation?<br>What costs and efforts are needed for communication and implementation?

10. **Key messages/lessons learned**	What key messages and lessons learned could be summarised from this story?<br><br>This can also be explored in a second interview, after writing the report and filling in the checklist.
11. **Available materials**	Are photos or video recordings available of the interventions? And a C action plan?<br><br>Also consider changed protocols or roadmaps, posters, photos of fun stickers or tools used to help keep the target group on track, etc.<br><br>Remember the consent form for photos and videos.
12. **Selection of communication channels**	Which communication channels are used to convey the message? Which media and tools are used to inform the target group?<br><br>Examples include:<br><br>- the website of the sector organisation,<br>- YouTube videos,<br>- own website,<br>- lesson materials, etc.
13. **Who is it for / which sectors are relevant?**	Which other settings have similar characteristics and can benefit from this example?
14. **More information**	Additional comments and/or important information?

Supplement S2. Analysis tool (Code tree)

Checklist for light, inspiring examples – state main theme, subtheme and setting here in the main line, along with a number of keywords that clearly show what this example is about and what other themes it is related to

Name of example	Description of intervention, solution or tool	Target group	Key question/specific details
Measures	Intervention/solution focuses on	Determinants/effects	Dilemmas/issues related to quality of life
General			
Staying 1.5 metres apart			
Social distancing			
Hygiene			
Face mask			
Testing behaviour			
Quarantine			
Vaccination			
Other	Context	Communication	Check/learn
Active ingredients and possible pitfalls (self-reported)			
Key messages, For inspiration			
Argumentation			

Checklist for light, inspiring examples – state main theme, subtheme and setting here in the main line, along with a number of keywords that clearly show what this example is about and what other themes it is related to

Why interesting/inspiring? Doubts?	
Judgement of the test committee	
Communication (photos, images)	

Supplement S3. Definition of a narrative

….Based on the work of Barbara Czarniawska [8] we use the following definition of 'a narrative':

'…a written text giving account of an event/actions or series of events/actions, chronologically connected and were people give information about their intentions, purposes, interactions with others and reflect about the meaning of their actions or behaviour in a certain period of time' (Czarniawska, p.6-12).

We based our understanding of 'narratives' mainly from Barbara Czarniawska (see ref.10) as a rich form of understanding the social world, … 'as a mode of knowing and communication' (page 6 to 12). Czarniawska explains narrative as a form of hermeneutic studies.

With the collection of narratives (based on 65 interviews) from 32 different contexts and settings we aim at gaining deeper insight in the behavior of people in pandemic times and of the meaning that people give to their behavior in their specific context. With a narrative we make it easy for readers to empathize with the respondents situation, to dive into their world (as far is possible) and learn from the key lessons and findings. In the analysis we specially search for context specific and common overall insights.

Interesting and relevant is the next quotation concerning de character of a narrative with a plot (by Todorov, 1971): 'An ideal narrative begins with a stable equilibrium which is disturbed by some power or force. There results a state or disequilibrium; by the action of a force directed in the opposite direction, the equilibrium is re-established, the second equilibrium in similar to the first, but the two are never identical (Czarniawska, p.19)'.

Supplement S4. Translated narrative: Free Food Market and background info

Free Food Market: in or out of the shadows?

Helen X , community member and driving force behind Helen's Free Food Market in the Vogelbuurt area of Amsterdam North, never expected her food market to draw as many people as it has. What began as a spontaneous initiative against food waste has grown into an indispensable lifeline for the most vulnerable people in the neighbourhood. With the pandemic dragging on, this form of 'social shadow work' is filling a gap between the informal and official forms of assistance.

The interviews were conducted in February 2021.

For this story we spoke with Helen X, who launched the initiative, as well as Jeanette X, community development worker with Diaconie Noord, and Saskia X, senior researcher in the Urban Social Work research group at the Amsterdam University of Applied Sciences (HvA) and co-author of this story.

Free Food Market as a phenomenon

Helen's Free Food Market (HFFM) started small, and – just like similar initiatives in many other communities in the Netherlands – arose from a personal interest in taking action against the tremendous volumes of food waste. "I was deeply concerned that there was still so much food being thrown away, when people around the world are still going hungry", Helen recalls. "By starting the food market, which I launched in summer 2019 with another local resident, we were trying to do something about it. This Amsterdam city district is home to lots of people who are undereducated and don't have much money. Some extra food makes a real difference in their lives."

The fact that her Free Food Market had become an indispensable resource for vulnerable people just one year later was also related to the coronavirus pandemic – although it did initially look like the lockdown might mean the end of her initiative due to safety concerns. "During the first lockdown, we had to change things fast", Helen says, "switching from a food market where people could pick up food for free to a grocery delivery service. Some people didn't even dare to leave their homes, that's how scared they were. And we didn't want the market to become a source of infection, even though we were among the first to wear face masks and ensure distancing. But we saw right away that there were tremendous amounts of food going to waste. All the restaurants, and a little later the wholesalers too, had so much food and no customers."

Background (this information can be unfolded on the website)

There are more than one million people living below the poverty line in the Netherlands (Statistics Netherlands, 2018). Official food banks and informal food initiatives help the people who need assistance the most by providing temporary food packages at no cost. In many cases, a food project begins as a small private initiative and later expands to become a full-fledged food bank; if they meet the conditions, they can join the national association of food banks. That organization now oversees 171 food banks with 10 distribution centres. In 2020, 160,500 people obtained food assistance through one of these official food banks. These efforts rely on partnerships between various organizations, governmental agencies and private parties. Some 13,000 volunteers are engaged in organizing and distributing the food. [1]

Facebook as a trigger

It was only when Helen posted a Facebook message and attracted media attention that she really came to understand how much her initiative filled a serious need. "The registrations just kept coming in", she recalls. "The delivery service, which I got off the ground as fast as I could with the help of the Sorgbasis initiative, was the answer." With a few cargo bikes and a handful of volunteers, the groceries could get to the people who needed them. "We had no trouble finding volunteers; so many people in the community were sitting at home and eager to do something good."

Free food to feel good

Helen has seen the target group of people who need food shift over the past few years. "Before the pandemic, it was primarily the low-income members of this community, people who had always lived here, along with people who believed in fighting food waste", she says. "But during the first lockdown, we saw more and more people with an immigration background coming in. Undocumented people are now also part of our target group, though this is not something we can prove, because we don't check. These are mainly people who don't have the right papers to access the official food bank, people who were previously doing informal work with no guarantees. All that work is gone now."

Gradually, Helen says, she also started seeing more freelancers, small business owners and students. "For these people, the step to a regular food bank is much bigger. Shame is a real factor here." By maintaining an emphasis on the Free Food Market's original concept of fighting food waste, Helen has helped people accept the help they need. "Last year we rescued over 20,000 kg of food that would otherwise have been thrown away", she says. "Without our customers, that food would have had nowhere to go. I'm trying to make it a feel-good experience. People feel like they're doing something for the planet while benefiting themselves as well."

Background (to be unfolded)

The number of people using the official food banks has increased, due in part to the COVID-19 pandemic. The association for food banks in the Netherlands reports that the number of households using food assistance has risen by 7.2% (reference date 31 December 2020). Amsterdam has not only seen a surge in the use of official food banks; the number of informal food banks has also spiked, MUG magazine reported in early 2021.

At present, food assistance offered through informal channels is reaching about 24,000 people at 70 locations in the city. This includes all the food initiatives set up by independent organisations, charitable institutions, local residents, and church-based groups. Here, too, the COVID-19 pandemic is considered the cause of the major increase in alternative food banks. Undocumented people are said to represent a majority of those served by these facilities (75%).

Meanwhile, a survey of participants at seven food banks in the nearby city of Utrecht showed that many people who rely on food assistance report striving for a balance between 'giving and taking' and reciprocity [2]. The researchers reported that many of those surveyed were embarrassed about relying on food bank assistance, and struggled to maintain self-respect. These people emphasized that they wanted to do something in exchange for the free food package. For many of them, this took the form of volunteering with the food initiative.

Out of the shadows

A sharp-eyed official of the municipality of Amsterdam caught wind of HFFM. "The area coordinator paid us a visit, and was enthusiastic about what he saw", Helen says. "He said, 'I see people here who I don't see at the regular food bank.'" After six months of dialogue, the Free Food Market was

able to go forward as an authorised facility with municipal support. "But with the commitment from them that I could continue using my own approach, staying autonomous and keeping the focus on the groups in this community who really need it", Helen says. The initiative to establish a dialogue between all food initiatives in Amsterdam North, the municipal authorities and official care systems came from Diaconie Noord, a volunteer platform for community engagement operated by the deaconate ministries of several Protestant churches in that part of Amsterdam. Other organizations, including the regular food bank, Human Aid Now and the Red Cross, soon came on board. This created a strong network supporting food assistance, and helped them pinpoint the people who need it most.

Social shadow work

The Free Food Market can be seen as a form of 'social shadow work', in which informal assistance (in many cases initiated by active locals) fills the gaps left in the official assistance infrastructure. "COVID-19 increased the need for this kind of assistance", says Saskia Welschen, whose work at the Amsterdam University of Applied Sciences has long focused on researching the phenomenon of 'social shadow work' and the relationship between informal and official facilities and initiatives. "That's not to say that official facilities are not successful, but what is clear is that they are not completely succeeding in answering the needs of some people."

HFFM reveals that new groups of people are now struggling as a result of the COVID-19 pandemic. People who would normally be just barely managing to cover their basic needs are now falling below the minimum subsistence level. "And it's these target groups in particular who feel the need to stay in the shadows, often out of shame or because they are undocumented", Saskia says. "But many of these people have also had bad experiences with official assistance and are suspicious of institutions. The people running the informal facilities sometimes succeed in reaching these groups and bringing those people's assistance needs out into the open. Cooperation between informal and official facilities can ensure that people can be channelled towards the right assistance. But it's important to tread lightly, because you can't risk losing their trust."

Helen wholeheartedly agrees with that last point: "I am very careful and never ask for personal information. If people tell me in conversation that they need help, then I always first ask whether I can put them in contact with an official institution. And I report the trends that I see to the municipality. But no specifics, just general trends. People need to know that they're safe with me."

Background (to be unfolded)

Since the decentralisation of the welfare state and the implementation of the Social Support Act (Wet Maatschappelijke Ondersteuning, WMO) in 2015, there has been a strong emphasis on individual resilience and self-sufficiency, and the degree to which they can offer each other support within social networks. This takes place within the 'informal domain', ranging from active locals, churches and mosques to migrant 'self-organisations' and sports teams.

Informal actors often initiate food projects, sometimes for reasons other than food assistance. In recent years, the Amsterdam University of Applied Sciences has studied [3-6] the role that these informal actors play as sources of care and support, and specifically for local residents who may be reluctant or unable to access official care and welfare facilities, for various reasons.

Many of the insights from this research are reflected in the example of Helen's Free Food Market. For example, informal initiatives are often perceived as low-threshold and are not based primarily on providing assistance. These initiatives can be seen as a form of social shadow work [7]. They succeed in winning trust through their approach, their first-hand knowledge and their proximity to the target group. In this role, they can act as bridges between distrustful local residents with a need for assistance or information and the official facilities.

On the other hand, the interaction between the official and informal domains is not without its own tensions, and establishing connections with the official apparatus is not always an objective

of the actors in the informal domain. We can also see aspects of this in the example of Helen's Free Food Market. Unfamiliarity, or even mistrust, between the two domains can play a role. This also applies to the relationship between informal actors and the government. Informal actors frequently feel that their contributions are not being sufficiently acknowledge and would like to see more support in this regard. At the same time, that support cannot come at the cost of their autonomy, which is what enables them to maintain their own approach.

Measures that do not make the work any easier

"COVID-19 brought people to the Free Food Market, but they are telling us that following the coronavirus measures, and sticking to them, is an extra burden on people who already had it tough before the pandemic", says Jeanette, a community development worker with Diaconie Noord. As a community organiser, networker and supporter of numerous poverty-related initiatives in the neighbourhood, she saw how the 1.5-metre distancing requirement caused many problems. "I've seen a lot of food initiatives arise, but most were organised from a house or a garage box. Not much room, of course, and it's really impossible to keep distancing, not least because people know each other and treat each other 'like family'. I've been engaging with them about this issue."

Jeanette says that especially at the start of the pandemic, a lot of people didn't understand how serious it was. On the one hand there were people who panicked and were too afraid to leave their homes, becoming extremely isolated; on the other, there was the group that didn't seem to pay attention to the measures at all. "They didn't see anyone in their communities getting sick, so they thought it wasn't that bad, or that it was just a flu", Jeanette explains. "That's different now, because a lot of people living here got COVID-19, and then it sank in. But I only started to see more people wearing face masks here once it was really mandatory."

Jeanette says that one important factor among the group that does not take the measures seriously is that these people have already been living in poverty for many years; they are used to a complex existence full of problems and often had to go without even the basic necessities. "At that point, a virus doesn't make much difference, and they won't let it scare them off", Jeanette says. What she says it takes to get them to take the measures to heart is generally a face-to-face discussion that really conveys what the measures are and why they are important. "Which is really too bad", says Jeanette, "because the government thinks that a press conference makes everyone understand, but that's really not the case. For us, that's only the start."

Background (to be unfolded)

Face-to-face contacts in a safe and informal setting often present good opportunities for preventive measures, because they can be an important way to identify needs. People who are having a hard time can be referred to assistance through official channels. In addition, informal key figures can play a role in communicating information about safety during a crisis, such as the coronavirus measures, and lead by example. This is why it is important for municipal authorities and these key figures in districts and neighbourhoods to understand and acknowledge this role.

One example of another form of informal assistance to people who are struggling with a lot of problems are the listening groups that have been launched by Diaconie Noord. These are small groups in which local residents meet to share their concerns. These listening groups were set up based on the Integrative Community Therapy (ICT) methodology. This is a preventive, communitybased mental health intervention developed in a Brazilian favela [8, 9]. This form of community therapy brings local residents together to share their day-to-day experiences, listen to each other and learn from each other. This initiative by Diaconie Noord answers a growing need for psychosocial support, because problems have been piling up throughout the pandemic for

many people living in this area. The groups are generally led by people who are already leaders in the neighbourhood or community.

Challenges in connecting the official and the informal

Saskia says that the Free Food Market is emblematic of what is needed, but also shows where the current system is failing. The COVID-19 pandemic continues to reveal new groups of the needy who do not meet the criteria for participation in the regular food bank system, and are therefore falling between the cracks. Informal and semi-informal facilities like HFFM are filling some of these cracks and becoming an essential part of the day-to-day support for large groups of vulnerable people. As such, they are more than an extension of the official assistance apparatus. This is due to the individual character and unique approach behind these initiatives.

The trick will be to acknowledge and support this valuable role and to ensure that the spheres of informal and official social work complement each other even better in future, while retaining the unique qualities of the informal initiatives, like the low threshold for people who mistrust the official channels. "The drivers, the community organisers, the innovators like Helen and Jeanette deserve every support we can give them, but they have to be able to keep their own specific values and keep reaching the people who really need that approach", says Saskia.

Tips & tricks

As an informal player or professional, be aware that you are always setting an example for the people you come into contact with, and that you can help them cope with behavioural measures during times of crisis. Most of all, remember that once built, trust is often fragile and can easily be broken.

Key messages

- By placing the emphasis on fighting food waste and the feel-good image of informal food initiatives, people who are unable to meet their own needs in a time of crisis can be helped across the threshold, thus mitigating the impact of poverty during a crisis.
- The low threshold and the room for privacy often enable informal initiatives to gain people's trust. In times of crisis, this is also a good basis for making contact with people and helping them cope with the measures.
- The challenge is to make sure that official and informal social work infrastructures complement each other to the maximum possible extent, while retaining the low-threshold approach and the added value that informal initiatives offer.

References:

1. Voedselbanken Nederland (2020). Feiten en Cijfers Voedselbanken Nederland – 2020.
2. Kromhout, M. & L. van Doorn (2013). Voedselbanken in Utrecht. Deelnemers in beeld. Utrecht: Utrecht University of Applied Sciences, Research Group on Innovative Social Services.
   https://www.internationalhu.com/research/innovative-social-services
3. Veldboer, L., & Hoijtink, M. (2019). "Hybridisering van wijkteams en vrijwilligersinitiatieven lijkt nog ver weg". Socialevraagstukken.nl.
   https://www.internationalhu.com/research/innovative-social-services
4. Welschen, S., Lucas, P., Hoijtink, M., & Veldboer, L. (2020). Licht op sociaal schaduwwerk: Literatuurstudie naar (Nederlandse) informele sociaalwerkpraktijken die plaatsvinden onder de radar. Amsterdam University of Applied Sciences.

5. Welschen, S., Lucas, P., Von Meyenfeldt, L., Hoijtink, M., Rijnders, J., & Veldboer, L. (2020). "Toegankelijkheid in divers perspectief. Bewoners, verbinders en professionals over de sociale basis in een ontwikkelbuurt". Final report on accessibility of basic facilities in Geuzenveld, Amsterdam. Amsterdam University of Applied Sciences, Werkplaats Sociaal Domein Amsterdam en Omgeving.
6. Welschen, S., Metze, R., Haijen, J., & Rijnders, J. (2018). "Met elkaar of naast elkaar? Wijkteamprofessionals en informele krachten in Oud Noord over kwesties bij de gezamenlijke ondersteuning aan huishoudens in kwetsbare posities." Amsterdam University of Applied Sciences.
7. Schrooten, M., Thys, R., & Debruyne, P. (2019). Sociaal schaduwwerk. Over informele spelers in het welzijnslandschap. Brussels: Politea.
8. Thys, R., Branca Prado, C., & Brandão Cruz, A., "Een Brusselse zelforganisatie voor gemeenschapstherapie of AETCIS. Een schoolvoorbeeld van 'vermaatschappelijking van de zorg'". In: Schrooten, M., Thys, R., & Debruyne, P. (2019). Sociaal schaduwwerk. Over informele spelers in het welzijnslandschap. Brussels: Politea. p.80-86.
9. Bonilla, A. (2008). "Revitalizing the Human Spirit Together: A Case Study of Movimento de Saúde Mental Comunitária do Bom Jardim in Bom Jardim, Fortaleza, Ceará." Independent Study Project (ISP) Collection 3, https://digitalcollections.sit.edu/isp_collection/3. https://core.ac.uk/reader/232724690

The interviews were conducted in February 2021.
